# Crosson (D. M.) on Anomalous Ducts of Exits from the Parotid Glands in Front of the Ear

**Published:** 1888-10

**Authors:** 


Crosson (D. M.) on Anomalous Ducts of Exits from the Parotid Glands in front of the Ear.—I was called to see the children of Mr. D., a very wealthy farmer, six miles distant in the country. I examined the children (four in number), and found that three of them have a small orifice on each side and just in front of the upper portion of the external ear, and that one has only one opening in front of the left ear, and that there is a continuous discharge going on through these orifices all the time. On close investigation, I diagnosed these openings to be mouths of small ducts leading from the parotid glands, and that, instead of the secretions being poured out through Steno's ducts at the proper place, they are discharged through these ducts externally, just in front of the external ear. Mr. D. has three children that have not this abnormality, but he has a brother-in-law (a brother to Mrs. D.) who has two children hat have the like ducts discharging externally—making, in all, six cases, four males and two females, in the same family. I find that whenever the excretions from the parotid glands through those ducts come in contact with the air in very cold weather, and when the mouths of the ducts are not kept clear and open to give free exit to the glandular secretions, the secretions accumulate in the ducts and produce glandular irritation, and when opened externally, discharge a very purulent excretion. I opened up two of the ducts in each of two of the children, which were blocked up, and these discharged considerably. I probed two of these ducts with a small silver probe, and found that they led directly into the parotid glands. I advised the parents to keep the ducts open to give constant and free exit to the secretions, and they have no trouble when this is done; and the discharge, when constantly going on, is so small that it is seldom noticed when kept clean—*Vir. Med. Mo.*

